# Protective effect of Secukinumab on severe sepsis model rats by neutralizing IL-17A to inhibit IKBα/NFκB inflammatory signal pathway

**DOI:** 10.1186/s40001-022-00845-2

**Published:** 2022-10-17

**Authors:** Xingsheng Wang, Xinxin Zhang, Li Sun, Guangsheng Gao, Yun Li

**Affiliations:** 1 Intensive Care Unit, Central Hospital Affiliated to Shandong First Medical University, Jinan, Shandong China; 2 Department of Emergency Medicine, Fuyang People’s Hospital, Fuyang, Anhui China; 3 Intensive Care Unit, Jinan Central Hospital, Cheeloo College of Medicine, Shandong University, Jinan, Shandong China; 4 Central Hospital Affiliated to Shandong First Medical University, NO. 105 Jiefang Road, Jinan, 250000 Shandong China

**Keywords:** Secukinumab, Sepsis, Acute lung injury, IL-17A, Inflammatory pathway

## Abstract

Secukinumab is a specific neutralizing antibody for IL-17A. At present, numerous studies have confirmed the important role of IL-17A in sepsis, but the role of secukinumab in sepsis has not been studied. The present study explored the protective effect and underlying mechanism of secukinumab in severe sepsis model rats. We established a severe sepsis rat model using cecal ligation and puncture (CLP). The optimal dose of secukinumab was determined by observing the 7-day survival rate of severe sepsis model rats. The expression levels of TNF-α, IL-6, and IL-17A in plasma and lung tissue were determined by enzyme-linked immunosorbent assay. The degree of pathological damage to lung tissue was evaluated by hematoxylin–eosin (H–E) staining and pathological damage scale. The expressions of IKBα/NFκB pathway proteins and downstream-related inflammatory factors were detected by western blotting and real-time quantitative polymerase chain reaction (RT-qPCR). Our results show that high-dose secukinumab can inhibit the activation of the IKBα/NFκB inflammatory pathway by neutralizing IL-17A and reducing the gene expression of pathway-related inflammatory cytokines, thereby reducing the levels of inflammatory cytokines in lung tissue and plasma, thereby reducing the damage of lung tissue in severe sepsis model rats and improving the systemic inflammatory response.

**Supplementary Information:**

The online version contains supplementary material available at 10.1186/s40001-022-00845-2.

## Introduction

Sepsis is a vital medical health problem, that affects more than tens of millions of people worldwide each year, with an estimated global mortality rate of over 25%. Although current guidelines on the treatment of sepsis are continuing to improve, the incidence of sepsis in the past 10 years is still on the rise, and the sepsis-related acute lung injury mortality rate remains as high as 70% [1–4]. Therefore, alleviating acute lung injury caused by sepsis is extremely important to reduce sepsis mortality. More than 100 clinical trials have been conducted on more than 100 biological agents, including tumor necrosis factor (TNF) and interleukin-1 (IL-1) inhibitors, for the treatment of sepsis, but no specific drugs have been found at present [5, 6]. Therefore, it is of great significance to explore new drugs and therapeutic targets that can effectively treat sepsis.

IL-17A, the first member of the IL-17 family to be discovered, was cloned in 1993 and was originally named cytotoxic T lymphocyte antigen-8 [7]. Patients with acute lung injury caused by sepsis have been showing consistently high levels of IL-17A. In the lipopolysaccharide (LPS)-induced acute lung injury (ALI) mouse model, increased IL-17A level was closely correlated with the severity of ALI [8, 9]. J Li and L Li et al. [10, 11] also confirmed in their respective studies that the high expression of IL-17A in the abdominal cavity plays a key role in the strong and sustained inflammatory response after severe sepsis, but by neutralizing IL-17A in the abdominal cavity, the production of pro-inflammatory cytokines can be reduced and neutrophil infiltration and lung injury can be alleviated. This suggests that IL-17A may be a therapeutic target for sepsis-associated acute lung injury.

Secukinumab is a high-affinity fully human monoclonal antibody expressed in a Chinese hamster ovary cell line (CHO-HPT1). It belongs to the IgG1/κ isotype subclass and selectively binds to IL-17A and neutralizes the bioactivity of this cytokine. Secukinumab has shown efficacy in key clinical areas of psoriasis and ankylosing spondylitis [12–14], but there is still a lack of relevant research on the role of secukinumab in sepsis. In this study, we found that high-dose secukinumab protects rats with severe sepsis by neutralizing IL-17A and inhibiting the IKBα/NFκB inflammatory signaling pathway.

## Materials and methods

### Experimental animals

SPF male SD rats, 6 weeks, 220–240 g, were purchased from Speifu (Beijing) Biotechnology Co., Ltd., and were adaptively reared for 1 week in the SPF Experimental Animal Center, Central Hospital Affiliated to Shandong First Medical University. The temperature is 23 ± 1 °C, the ambient humidity is 50 ± 5%, and a light–dark cycle is carried out every 12 h.

### Establishment of model and intervention

The rat model with severe sepsis was established by cecal ligation and puncture. Anesthesia was performed with 1% sodium pentobarbital (40 mg/kg). A longitudinal median incision was made on the skin of the middle and lower abdomen of the rat to enter the abdominal cavity. Blunt separation of the cecum, and ligated with 4–0 suture from the distal cecum to three quarters of the ileocecal valve. Using an 18G needle to puncture the cecum twice. Return the processed cecum to the abdominal cavity. Suture, and re-sterilize the surgical area with iodophor. The rats were resuscitated by subcutaneous injection of preheated normal saline (37 °C, 3 ml/rat). The sham operation group did not have the steps of cecal ligation and perforation. The cecum was bluntly separated and then placed back into the abdominal cavity. After the establishment of the sepsis model, the rats in the drug group were immediately given the corresponding dose of secukinumab by intraabdominal injection.

### Experimental grouping

First, a survival experiment was performed to examine the effect of different doses of secukinumab on the survival rate of severe sepsis model rats. Fifty rats were divided into the Sham group, CLP group, CLP + Sec (5 mg/kg) group, CLP + Sec (10 mg/kg) group, and CLP + Sec(20 mg/kg) group by completely random method, 10 rats in each group. Mortality was observed daily for 7 consecutive days. Then, the best dose (20 mg/kg) for improving the survival rate of rats with severe sepsis was used to explore the mechanism. Thirty rats were divided into Sham group, CLP group, and CLP + Sec (20 mg/kg) group by completely random method, with 10 rats in each group. 24 h after the establishment of the model, the rats in each group were killed under anesthesia, and the plasma and lung tissue samples were collected.

### Morphological observation of abdominal organs in animals

Abdominal viscera were fully exposed before specimen collection, and the morphology of abdominal organs of rats in each group was observed with naked eyes.

### Histopathological examination

Lung tissue was processed using standard methods. Briefly, the HE-stained paraffin sections of lung tissue were prepared through the steps of sampling, dehydration, waxing, sectioning, and staining. Microscopic examination was performed using a light microscope, and images were collected for analysis.

A method similar to that of YC Li et al. [15] was adopted to score lung tissue injury by professional pathologists under double-blind conditions according to the structural integrity of the alveolar cavity, and infiltration of neutrophils and lymphocytes, interstitial edema of the alveolar cavity and formation of the hyaline membrane. The scoring criteria were: no lung tissue damage, 0 points; lung tissue damage range  < 25%, 1 point; lung tissue damage range of 20–50%, 2 points; lung tissue damage range of 50–75%, 3 points; lung tissue damage range  > 75%, 4 points. Five different visual fields were selected for each pathological section for scoring, and the results were statistically analyzed.

### Lung wet/dry weight ratio

Part of the lung tissue was weighed using a microbalance and recorded as wet weight, placed in a 60 °C oven for 72 h, until the weight remained unchanged, then taken out, weighed again using a microbalance and recorded as dry weight, and the lung wet/dry was calculated weight ratio.

### ELISA

The levels of inflammatory cytokines IL-6, IL-17A, and TNF-α in rat plasma and lung tissue were detected using commercially available ELISA kits (Boster Bioengineering Co., Ltd., China). The absorbance (OD value) was measured at 450 nm using a microplate reader, the standard curve was drawn using ELISA Calc software, and the concentration of the sample was calculated.

### Western blotting

In short, the protein was extracted from lung tissue after homogenization, and the total protein concentration was determined by the BCA method. Protein samples were separated by sodium dodecyl sulfate–polyacrylamide gel electrophoresis and transferred to the PVDF membrane. After the transfer, the PVDF membrane was placed in a 5% BSA blocking solution and sealed with a shaker at room temperature for 2 h. PVDF membrane was inoculated with primary antibody (P-NF κB, NFκB, 1:1000; P -IκBα, IκBα, 1:10000; GAPDH and Tubulin, 1:50000; Abcam), 4 °C shaker overnight. The PVDF membrane was then placed in the secondary antibody and incubated in a shaker at room temperature for 1 h. Using ECL solution for imaging. Image J software was used to analyze protein bands.

### Real-time quantitative PCR

Gene expression of IL-6, IL-17A, TNF-α, IL-1β, and IFN-γ in rat lung tissue was detected using a fluorescence quantitative PCR detection kit (Yisheng Biotechnology Co., Ltd., China), and the operation was performed according to the instructions. Gene-specific primers are listed in Table 1.

**Table 1:** Gene-specific primers used for real-time RT-PCR

Gene	Forward primer	Reverse primer
TNF-Α	5ʹ-AGATGTGGAACTGGCAGAGG-3ʹ	5ʹ-CACGAGCAGGAATGAGAAGAG-3ʹ
IL-1Β	5ʹ-CTCGTGGGATGATGACGACC-3ʹ	5ʹ-AGGCCACAGGGATTTTGTCG-3ʹ
IL-6	5ʹ-TCCTACCCCAACTTCCAATGC-3ʹ	5ʹ-GGTTTGCCGAGTAGACCTCAT-3ʹ
IL-17A	5ʹ-CGCCGAGGCCAATAACTTTC-3ʹ	5ʹ-GGTTGAGGTAGTCTGAGGGC-3ʹ
IFN-Γ	5ʹ-GGCAAAGGACGGTAACACG-3ʹ	5ʹ-TCTGTGGGTTGTTCACCTCG-3ʹ
Β-Actin	5ʹ-CACCCGCGAGTACAACCTTC-3ʹ	5ʹ-CCCATACCCACCATCACACC-3ʹ

### Statistical analysis

Data analysis and drawing are carried out using SPSS 26.0 and GraphPadPrism 8.0 software. The quantitative data were expressed by mean ± standard deviation, the data of multi-group samples were compared by single-factor analysis of variance (ANOVA), and the data of the two groups were compared by double-tailed unpaired *t* test.

## Results

### Secukinumab can improve the survival rate of severe sepsis model rats

**Fig. 1:**
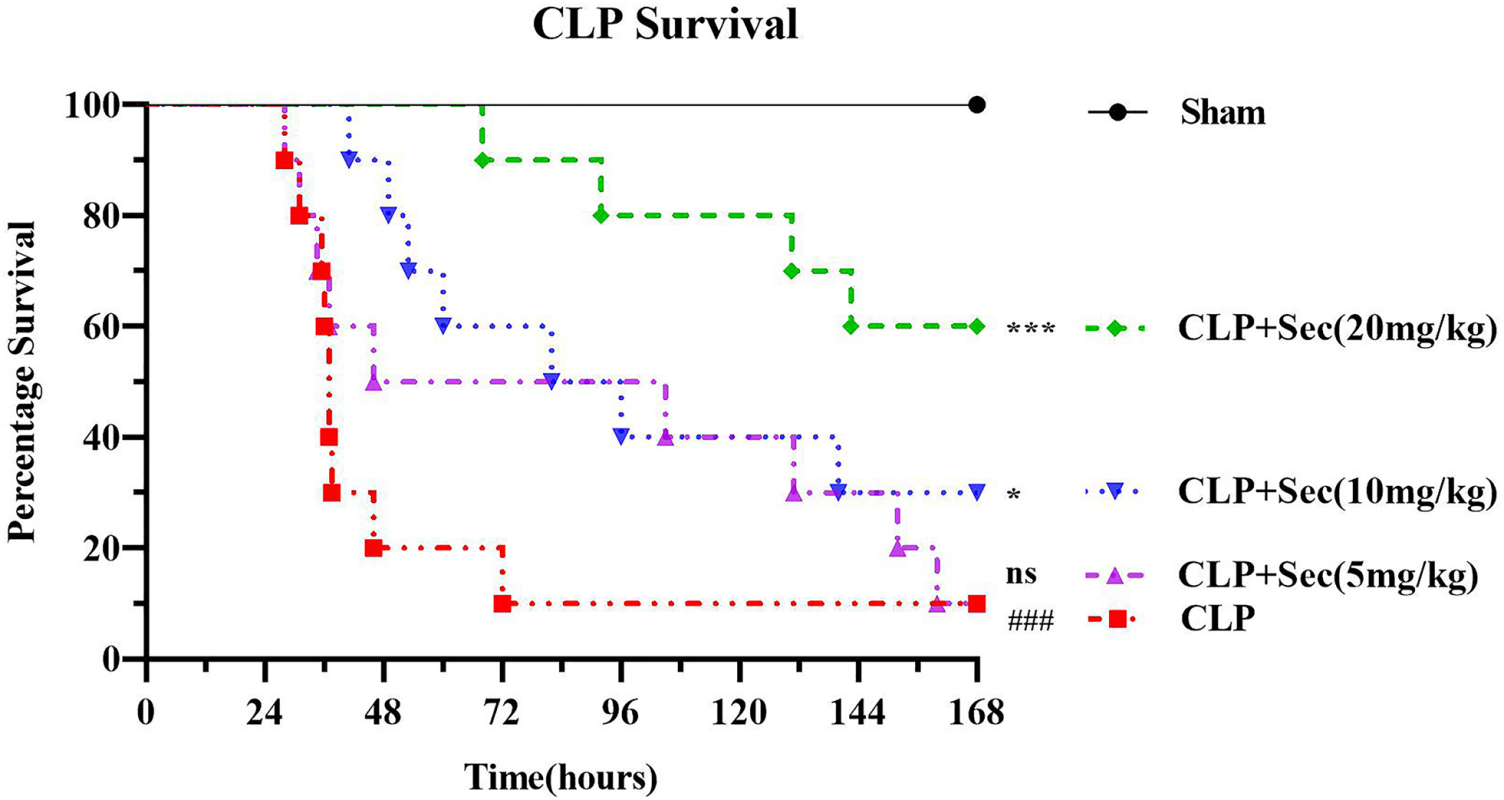
168 h survival curve of rats in each group. ns means *P* < 0.05 *vs* CLP, ###*P* < 0.001 *vs* Sham, *P < 0.05 *vs* CLP, ***P < 0.001 *vs* CLP, mean ± SD, one‐way ANOVA, double-tailed unpaired *t* test, *n* = 6/group

168 h after the operation, the rats in the Sham group did not die, and the survival rate of the rats in the CLP group decreased to 10%; the survival rate of the rats in the CLP + Sec (5 mg/kg) group was the same as that of the rats in the CLP group, and the survival rate between the two groups of rats was no difference (*P* > 0.05). Compared with the CLP group, the survival rate of rats in the CLP + Sec(10 mg/kg) group increased to 30%, while the survival rate of rats in the CLP + Sec(20 mg/kg) group was significantly increased to 60%, with statistically significant difference (*P* < 0.001) (Fig. 1). These results confirmed the protective effect of high-dose secukinumab on severe sepsis rats. Based on these results, we chose to use high-dose secukinumab (20 mg/kg) in subsequent experiments.

### Secukinumab can improve the morphology of abdominal organs in severe sepsis model rats

**Fig. 2:**
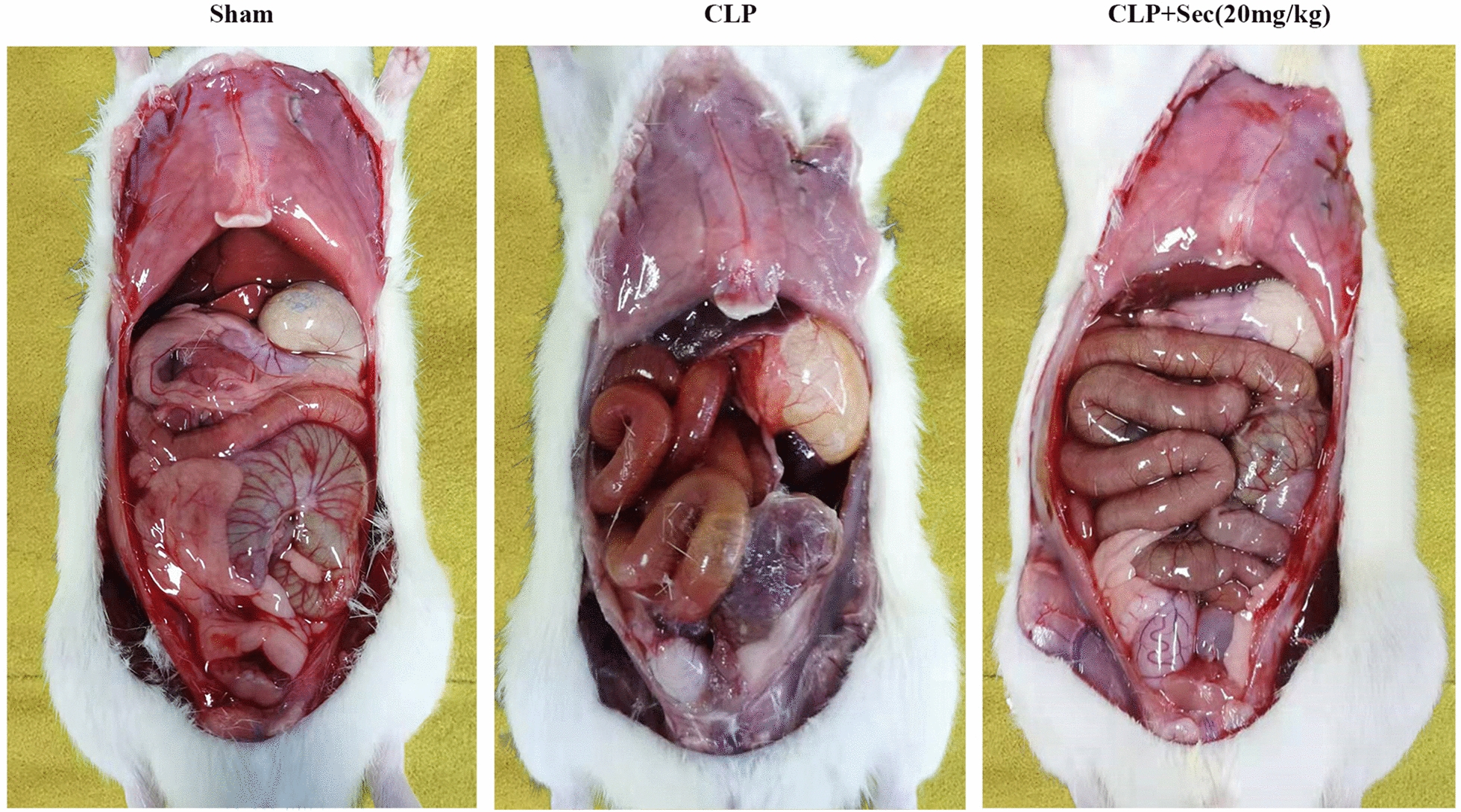
Observe the shape of abdominal organs of rats in each group with naked eyes at 24 h

24 h after the operation, the rats in the CLP group had obvious hyperemia and blackness in the liver, spleen, kidney, and other viscera, severe gastrointestinal edema and adhesion, a large amount of purulent fluid in the intestinal lumen, necrotic black caecum ligation, and a large amount of pus covering the surface. Compared with the CLP group, the degree of intraperitoneal organ congestion in the CLP + Sec(20 mg/kg) group was significantly reduced, the degree of gastrointestinal edema and adhesion and the degree of caecal necrosis of the ligated caecum were lighter, and the amount of purulent exudation around the caecum was less. (Fig. 2).

### Secukinumab can reduce the content of inflammatory cytokines in plasma and lung tissue of severe sepsis model rats

**Fig. 3:**
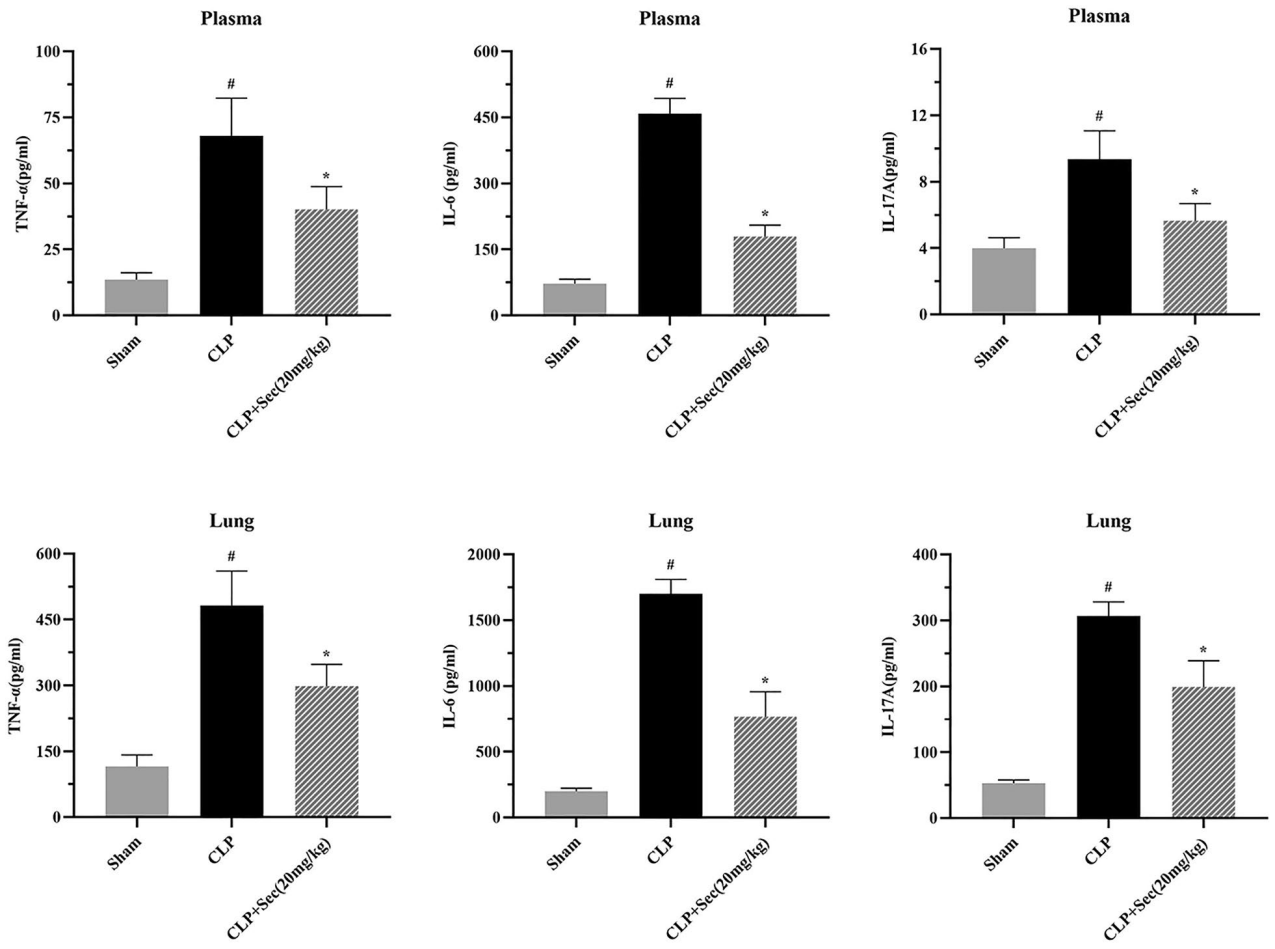
Contents of inflammatory factors TNF-α, IL-6, and IL-17A in plasma and lung tissue of rats in each group at 24 h. #*P* < 0.05 *vs* Sham, *P < 0.05 *vs* CLP, mean ± SD, one‐way ANOVA, double-tailed unpaired *t* test, *n* = 6/group

24 h after the operation, compared with the Sham group, the contents of inflammatory cytokines such as IL-6, IL-17A, and TNF-α in plasma and lung tissue homogenate of rats in the CLP group were significantly increased (*P* < 0.05). However, in the CLP + Sec (20 mg/kg) group, high-dose Secukinumab significantly reduced the levels of these inflammatory cytokines, with statistically significant differences between groups (*P* < 0.05) (Fig. 3).

### Secukinumab can protect against acute lung injury caused by severe sepsis

**Fig. 4:**
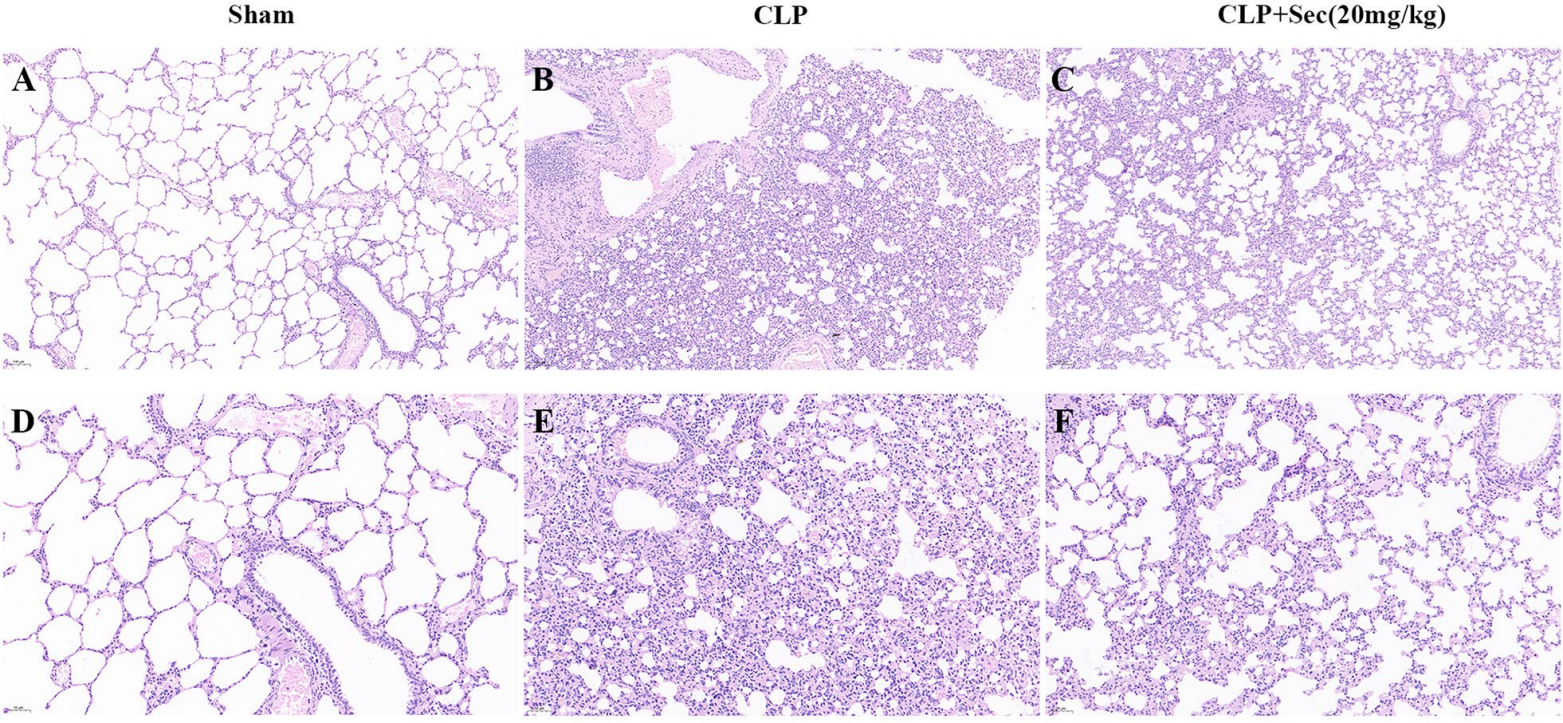
Lung tissue morphology of rats in each group under the light microscope at 24 h (HE staining). **A**, **B**, **C** Original magnification 100 × . **D**, **E**, **F** Original magnification 200 ×

**Fig. 5:**
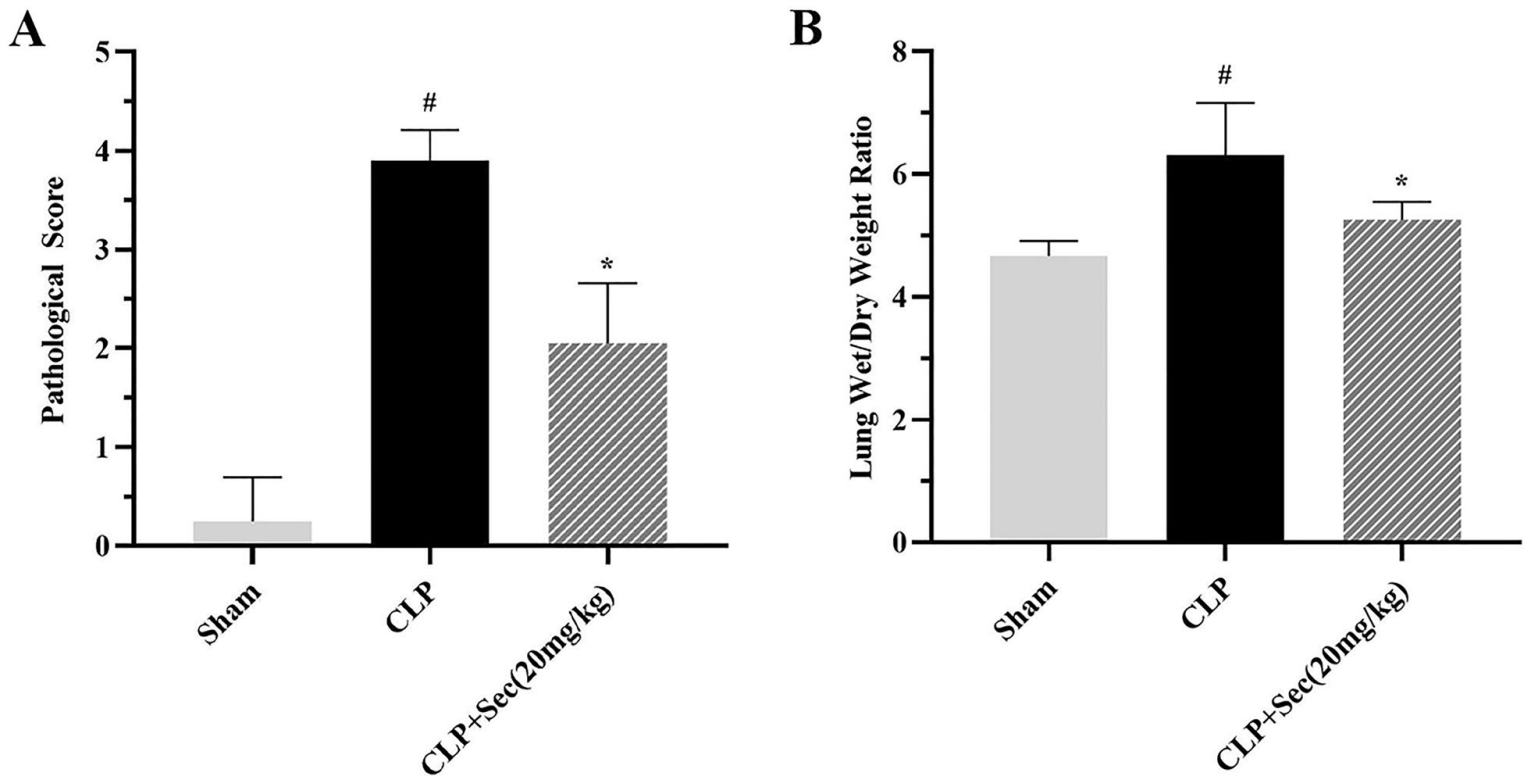
Lung histopathological score and wet/dry weight ratio of rats in each group at 24 h. **A** Lung histopathological score. **B** Lung wet/dry weight ratio. #*P* < 0.05 *vs* Sham, *P < 0.05 *vs* CLP, mean ± SD, one‐way ANOVA, double-tailed unpaired *t* test, *n* = 6/group

24 h after the operation, under the light microscope (200 ×), alveolar cavity structure destruction was observed in the lung tissues of rats in the CLP group, including alveolar cavity occlusion and consolidation, active fibroblast proliferation, destruction and thickening of the alveolar septum, and infiltration of a large number of neutrophils and a small number of lymphocytes in bronchiole accompanied by transparent membrane formation, and the lesions were diffuse. In the CLP + Sec (20 mg/kg) group, most of the alveolar spaces in the lung tissue of the rats were intact, with a small number of neutrophils and lymphocytes infiltrating the alveolar interstitium, and there was no obvious structural damage to the alveolar walls. Compared with the CLP group, the lung tissue pathological score was significantly decreased after treatment with Schuizumab, and the difference between the groups was statistically significant (*P* < 0.05) (Figs. 4, 5A). The wet/dry weight ratio of lung tissue mainly reflects the edema and inflammatory exudation of lung tissue. In the CLP + Sec (20 mg/kg) group, compared with the CLP group, secukinumab treatment significantly decreased the W/D ratio of lung tissue (*P* < 0.05) (Fig. 5B). These results suggest that secukinumab may have a protective effect on acute lung injury induced by severe sepsis.

### Secukinumab can inhibit the activation of the IKBα/NFκB pathway

**Fig. 6:**
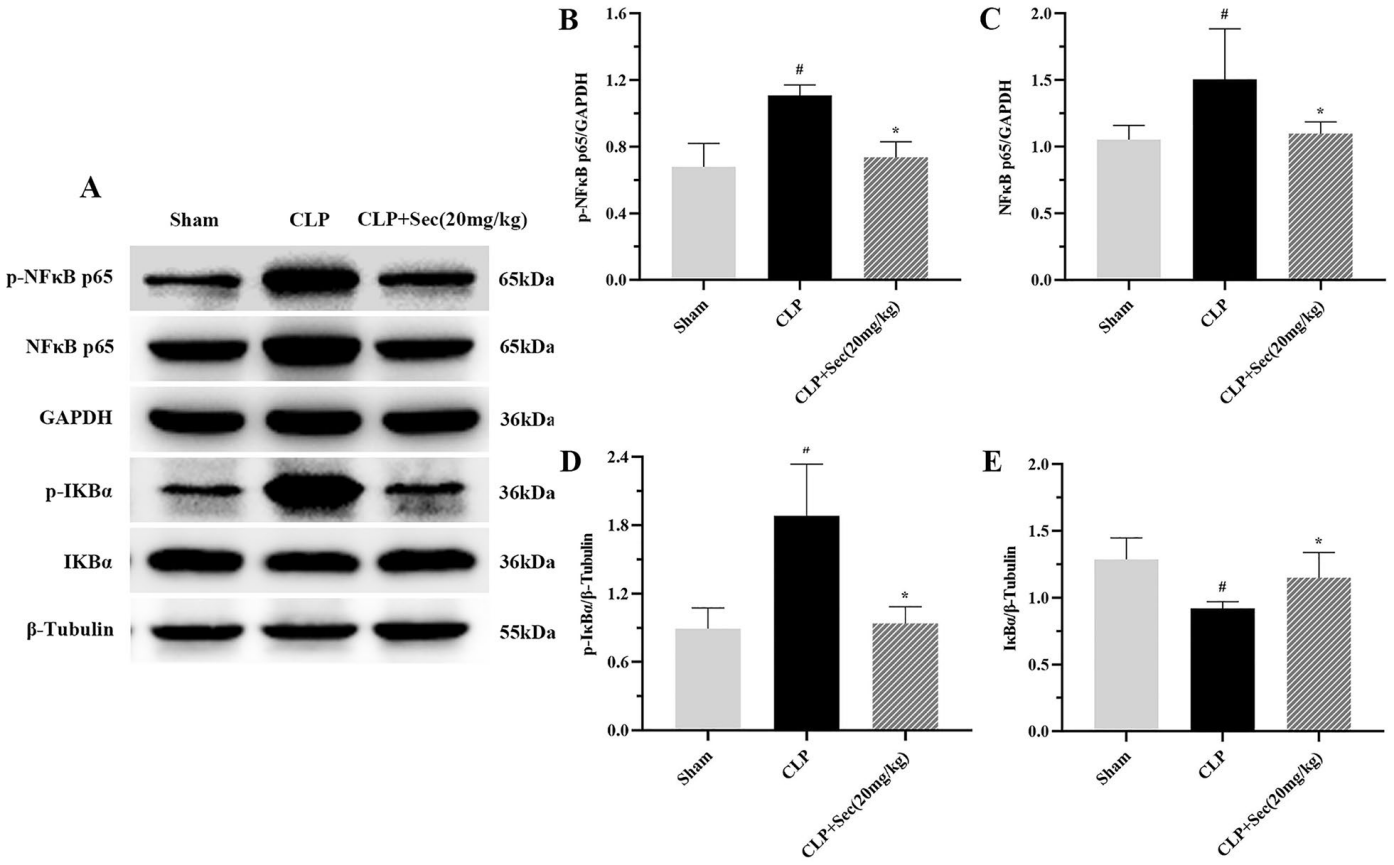
Protein expression of p-NFκB, NFκB, p-IKBα, and IKBα in lung tissue of rats in each group at 24 h. **A** Protein bands. **B**, **C**, **D**, **E** Protein expression levels. #*P* < 0.05 *vs* Sham, *P < 0.05 *vs* CLP, mean ± SD, one‐way ANOVA, double-tailed unpaired *t* test, *n* = 6/group

We used western blot to detect the protein expressions of p-NFκB, NFκB, p-IKBα, and IKBα to reflect the activation and activation of the IKBα/NFκB inflammatory pathway. Compared with the CLP group, the degradation and phosphorylation levels of IKBα in the CLP + Sec (20 mg/kg) group were significantly decreased (*P* < 0.05). Accordingly, compared with the Sham group, NFκB protein was increased and phosphorylation level was significantly increased in the CLP group (*P* < 0.05), while NFκB protein was significantly decreased in CLP + Sec(20 mg/kg) group compared with the CLP group, with statistical significance (*P* < 0.05). (Fig. 6).

**Fig. 7:**
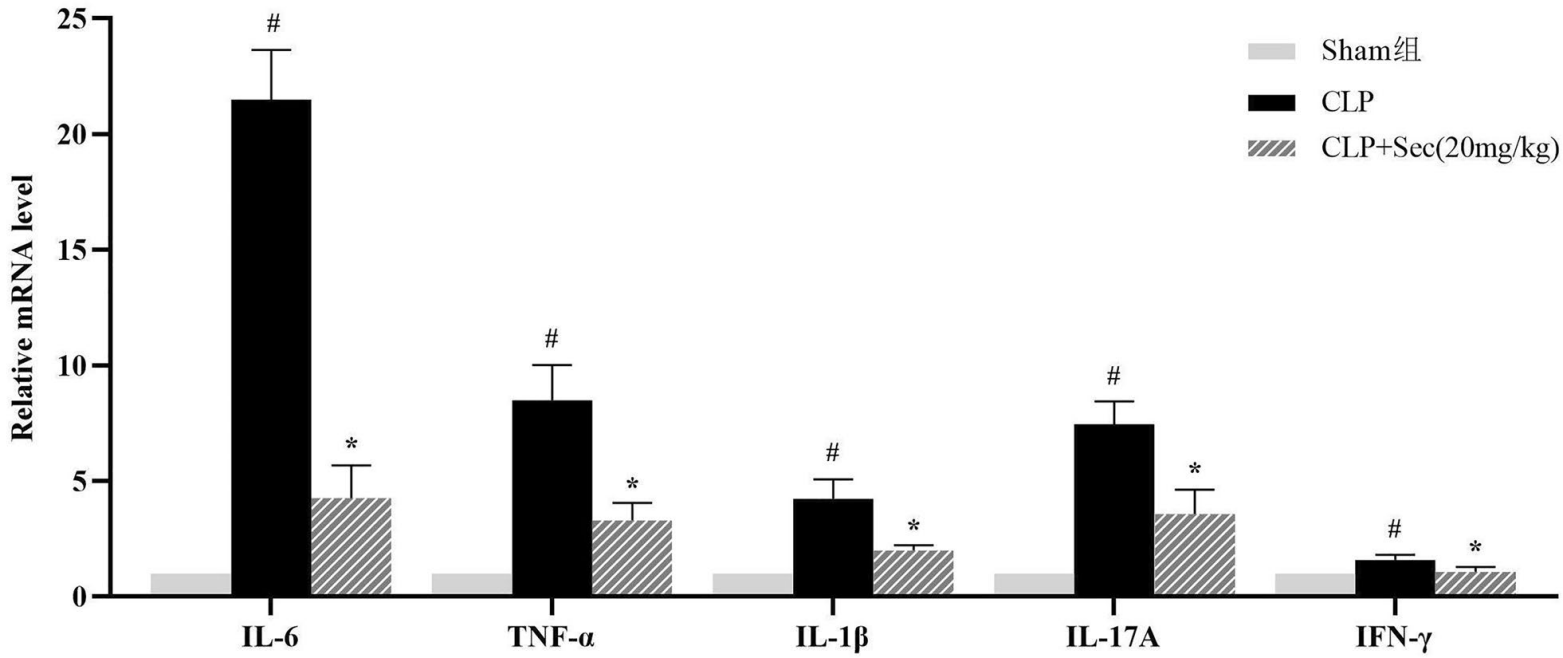
Gene expression of IL-6, TNF-α, IL-1β, IL-17A, and IFN-γ in lung tissues of rats in each group at 24 h (Fold = 2^−ΔΔCT^). #*P* < 0.05 *vs* Sham, *P < 0.05 *vs* CLP, mean ± SD, one‐way ANOVA, double-tailed unpaired *t* test, *n* = 6/group

RT-qPCR was also used to detect the mRNA levels of il-6, IL-17A, TNF-α, IL-1β, and IFN-γ downstream factors of the IKBα/NFκB pathway to reflect the role of secukinumab at the gene level. The gene expressions of IL-6, IL-17A, TNF-α, IL-1β, and IFN-γ were significantly decreased in CLP + Sec (20 mg/kg) group, with statistical significance (*P* < 0.05) (Fig. 7). Our results suggest that secukinumab may inhibit the activation of the IKBα/NFκB inflammatory pathway in severe sepsis by neutralizing IL-17A.

## Discussion

The IL-17 family is emerging as an important regulator of inflammatory response and has become a key to expanding the understanding of cytokine networks that coordinate innate and adaptive immunity [16]. The role of the IL-17A-related signaling pathway in sepsis is still a research hotspot. Roark CL et al. [17] showed that bacteremia significantly increased 6 h after the establishment of the non-severe CLP sepsis model in IL-17R knockout mice administered with neutralizing IL-17A. Flierl et al. [9] gave anti-IL-17A immediately after establishing a mouse model of severe CLP sepsis, and the 7-day survival rate of the mice increased to 58% compared with the 8% survival rate of the control mice treated with IgG, demonstrating that in the severe CLP model (the 7-day mortality rate was 100%) by neutralizing IL-17A successfully reduced mortality in mice. JB Li et al. [10] analyzed that in the severe CLP sepsis model, increased IL-17A produced by γδT cells induces neutrophil recruitment and promotes the inflammatory response, and subsequent studies proved that abundant IL-17A in the peritoneal cavity plays a key role in the intense and sustained inflammatory response of severe sepsis. Neutralizing IL-17A in the peritoneum has a protective effect on organ tissue damage and death caused by sepsis. Therefore, we speculate that the difference between these studies is due to the different severity of sepsis models used (Additional file 1).

Acute lung injury (ALI) is one of the early manifestations of sepsis, and its pathogenesis is still not completely clear. Some scholars believe that the destruction of the alveolar epithelium caused by sepsis is its pathophysiological basis [18]. QC Li et al. demonstrated a close correlation between increased IL-17 levels in LPS-induced ALI mice and the severity of ALI. Overproduction of IL-17 promoted the development of ALI, while IL-17 deficient mice were resistant to ALI induction. Mechanistically, IL-17 regulates lung inflammation in LPS-induced ALI mice [9]. Pulmonary edema is one of the typical symptoms of acute lung injury caused by sepsis [19] and is usually attributed to the loss of alveolar-capillary barrier structure [20]. The degree of pulmonary edema was evaluated by examining the W/D weight ratio of lung tissue in rats. Our results showed that the W/D weight ratio of lung tissue in rats with sepsis was significantly reduced by the neutralization of IL-17A, as well as the content of inflammatory factors, such as IL-6 and TNF-α. This is consistent with our lung histopathological results, suggesting that secukinumab can reduce alveolar epithelial cell edema and inflammatory exudation in the alveolar cavity of rats with severe sepsis.

The IKBα/NFκB inflammatory pathway is one of the most important inflammatory signaling pathways in cells, encoding many pro-inflammatory cytokines, such as IL-6, IL-1β, and TNF-α, which are involved in the gene expression of sepsis-induced inflammation and the pathophysiological process of ALI, and play a central role in regulating the inflammatory response [21, 22]. Inhibition of the IKBα/NFκB signaling pathway has previously been shown to play a protective role in sepsis [23]. Therefore, blocking the IKBα/NFκB signaling pathway can reduce the inflammatory response and lung injury in CLP-induced ALI mice. Monin L et al. [24] suggested that IL-17A and IL-17RA could bind and interact with NFκB factor activator 1(Act1), and then Act1 rapidly recruits and ubiquitizes TNF receptor-associated factor-6 (TRAF6), which triggers activation of the IKBα/NFκB pathway [25, 26]. In this study, we found that the expression of p-NFκB p65 and p-IKBα was significantly inhibited by the neutralization of IL-17A in the lung tissues of septic rats, and the expression of downstream genes such as IL-6, TNF-α, IL-1β, and IFN-γ was decreased by the neutralization of IL-17A. Based on these results, secukinumab may play a role in the treatment of sepsis-associated acute lung injury by inhibiting the activation of the IKBα/NFκB inflammatory pathway and thereby inhibiting the release of related inflammatory factors.

In addition to lung tissue, numerous studies have demonstrated that IL-17A levels are elevated during multiple organ injuries caused by sepsis. Experimental and clinical data of CJ Luo et al. [27] showed that IL-17A is associated with increased levels of pro-inflammatory cytokines in AKI and accelerated apoptosis of renal tubular epithelial cells. In addition, IL-17A can serve as a chemokine to recruit neutrophils to the kidney [28]. IL-17A is also upregulated in animal models of acute renal tubular injury and cisplatin-induced AKI [29]. The role of IL-17A has also been found in intestinal barrier dysfunction caused by sepsis. IL-17A-mediated inflammation disrupts intestinal epithelial barrier function, increases intestinal permeability, and leads to intestinal bacterial translocation by inhibiting intestinal cell proliferation and inducing its apoptosis [30, 31]. This is consistent with the results of our study. When dissecting septic rats, we found through naked eye observation that the intestinal tract of septic rats was dilated and edema, with severe adhesion, and a large amount of purulent fluid could be seen in the intestinal tract. Previous studies have shown that neutralizing IL-17A protects intestinal barrier integrity, reduces systemic inflammation and bacterial transmission, and reduces mortality in septic mouse models [32]. In addition, other experiments have shown that IL-17A levels are significantly increased in myocardial ischemia–reperfusion injury, inflammation, and apoptosis [33].

It is worth mentioning that although many previous studies have investigated the role of IL-17A in sepsis by neutralizing IL-17A with anti-IL-17A antibodies. However, our study is the first to demonstrate the protective effect of neutralizing IL-17A on rats with severe sepsis using an approved monoclonal antibody drug, Secukinumab.

There is increasing evidence that IL-17A is involved in the pathophysiology of sepsis, involving the regulation of inflammation and immune responses. Correspondingly, elevated IL-17A levels were associated with disease severity of sepsis, suggesting that IL-17A could serve as a potential biomarker for assessing prognosis in clinical settings. In addition, because IL-17A may be protective or pathogenic, the development of IL-17A as a therapeutic target must be situational. Future work will need to further explore the pathophysiological mechanisms of different immune responses during the neutralization of IL-17A in sepsis treatment.

## Conclusions

We found that the effect of a high dose (20 mg/kg) of secukinumab improved the survival rate in rats with severe sepsis induced by CLP for the first time. High-dose Secukinumab inhibits the activation of the IKBα/NFκB inflammatory pathway by neutralizing IL-17A, reduces the gene expression of pathway-related inflammatory cytokines, and thus reduces the levels of inflammatory cytokines in the lung tissue and plasma, thus alleviating lung tissue injury and improving systemic inflammation in rats with severe sepsis. Therefore, Secukinumab is a promising drug for the clinical treatment of septic acute lung injury, and appropriate treatment timing and dose may be the key to the successful treatment of these patients.

## Supplementary Information

**Additional file 1.** The complete protein bands.

## Data Availability

All data generated or analyzed during this study are included in this published article.
