# Supplementary material for: Protective effect of Secukinumab on severe sepsis model rats by neutralizing IL-17A to inhibit IKBα/NFκB inflammatory signal pathway
Source: Eur J Med Res. 2022 Oct 17;27:206. doi: 10.1186/s40001-022-00845-2 (PMC9578221; doi:10.1186/s40001-022-00845-2)
Supplement: Supplementary file 1 — Additional file 1. The complete protein bands. [file 40001_2022_845_MOESM1_ESM.pdf]

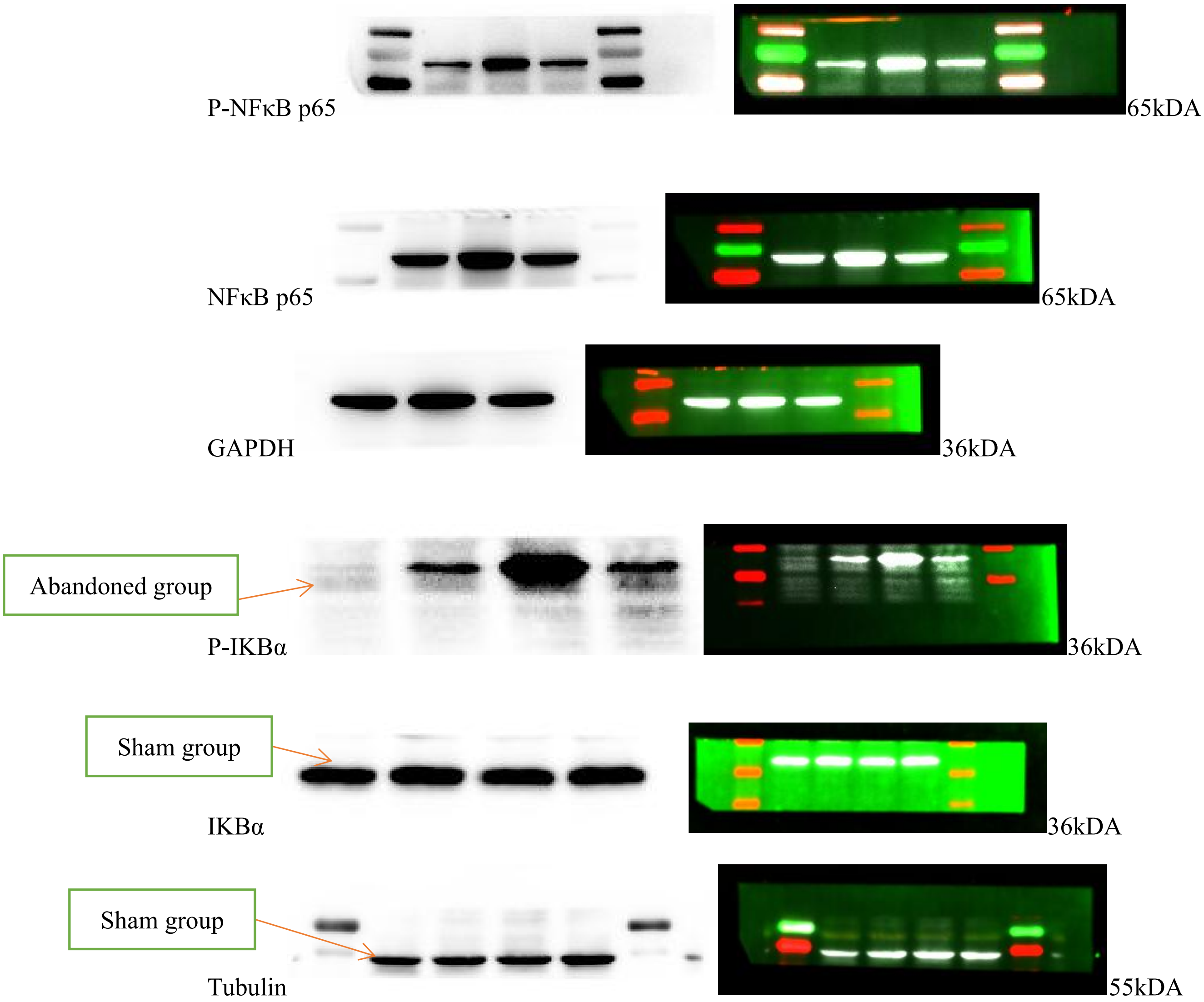

Means description of additional protein bands
